# Pituitary tumor centers of excellence for Cushing’s disease

**DOI:** 10.1007/s11102-022-01264-6

**Published:** 2022-09-10

**Authors:** Marcos Couselo, Stefano Frara, Andrea Giustina, Felipe F. Casanueva

**Affiliations:** 1 Santiago de Compostela University, IDIS—Complejo Hospitalario Universitario de Santiago (CHUS), CIBER de Fisiopatologia Obesidad y Nutricion (CIBERobn), Instituto Salud Carlos III, Santiago de Compostela, Spain; 2 Institute of Endocrine and Metabolic Sciences, San Raffaele Vita-Salute University and IRCCS San Raffaele Hospital, Milan, Italy

Pituitary tumors are more frequent than commonly thought [1, 2]. Despite the significant progress of pituitary sciences in the last years, some pituitary adenomas still present considerable challenges in diagnosis and treatment. Most hospitals present highly trained endocrinologists, pituitary surgeons, and other supporting specialists. However, their outcome is impeded by the fragmented approach to the problems. In most centers, specialists dealing with pituitary tumors are not integrated without networking activity and explicit protocols or validation by external auditors. In that situation appeared the need for the concept of Pituitary Tumors Centers of Excellence (PTCOE). The PTCOE involves a multidisciplinary team of experts in clinical neuroendocrinology, transsphenoidal surgery, radiodiagnostic, neuropathology, and other supporting specialists. Such teamwork in a center is integrated with standard protocols and group discussions, and the outcomes are reviewed and validated by external authorities [3].

Cushing’s disease is a complex disorder characterized by chronic hypercortisolism (Cushing’s syndrome) generated by a pituitary adenoma hypersecreting adrenocorticotropic hormone (ACTH). It is probably the most challenging pituitary adenoma with considerable problems in suspecting the disease, biochemical diagnosis, and effective treatment and follow-up [4].

When signs and symptoms of the patient suggest a Cushing’s disease, he first step is to demonstrate the presence of chronic hypercortisolism and then ACTH-dependent, which needs to be performed through biochemical tests [5, 6]. Clinical endocrinologists of the PTCOE need to have a deep understanding of the capability and mechanism of each of these tests. The first step includes urinary-free cortisol in 24 h urine collection (UFC), late-night salivary cortisol (LNSC), and a low-dose dexamethasone suppression test. At the PTCOE, endocrinologists work in close liaison with laboratory specialist experts in these tests and are well aware of the efficacy, limitations, confounding factors, and interferences [7–10]. No other pituitary adenoma needs so many tests for diagnosis, with the peculiarity that sometimes they need to be repeated and that frequently all of them need to be performed to reinforce the diagnosis. One of the major problems is to differentiate Cushing’s disease from non-neoplastic hypercortisolism. As the PTCOE is a patient-centered organization [3], the patients’ clinical history and interfering disruptors need to be carefully evaluated. The following step usually involves measuring ACTH either basal or stimulated by corticotrophin-releasing hormone (CRH) or desmopressin [11, 12]. It is easy to understand that such a complex biochemical diagnosis is favored by meeting discussions by laboratory experts and endocrinologists with the high experience attained in units with a high workload.

One of the basic supports of a PTCOE is the presence of a highly experienced neuroradiologist. After the biochemical diagnosis of Cushing’s disease, another challenging point is to localize the adenoma in the pituitary. The adenoma size in Cushing’s disease is variable. However, microadenoma is frequently difficult to visualize or is not visible under magnetic resonance imaging (MRI) [3, 13]. Even adenomas are frequently so small that with the standard 1.5 T MRI near 50% of microadenomas are not visible [13–15]. Positron emission tomography to detect Cushing’s disease adenomas is unavailable in many countries, and even results are variable [16, 17]. The collaboration with experienced neuroradiologists is also relevant in the cases where inferior petrosal sinus venous sampling (IPSS) was employed to differentiate Cushing’s disease from ectopic ACTH-dependent Cushing’s syndrome [18, 19]. As the combination of laboratory and image testing is necessary for the diagnosis of Cushing’s disease as well as to exclude ectopic ACTH-secreting tumors, this is only possible in highly specialized centers [20], being PTCOE the most logical structure for that task.

When considering the treatment, transsphenoidal surgery is currently the first-line therapy for patients with Cushing’s disease. The PTCOE [3] criteria put special emphasis on the presence of an experienced pituitary surgeon with in-depth training and who maintains expertise through a high workload [21]. The results of surgery for Cushing’s disease are highly effective in the hands of experienced surgeons [22–24]. Lower rates of positive results in the hands of less experienced surgeons are associated with a higher number of complications [22]. In any case, recurrences after pituitary surgery are possible. This caused mandatory lifelong follow-up and monitoring of the patient by the endocrine unit.

The role of the clinical endocrinology section on the PTCOE is complex, from clinical suspicion to disease control route. In the initial steps of management, and during follow-up endocrinologists are in charge of dealing directly or relying on expert consultants with the multiple complications of the disease, such as diabetes mellitus [25], hypertension, dyslipidemia, hypercoagulability, cardiovascular disease, bone comorbidities [26], and other pituitary hormones deficiency [27].

This complex task may be continued in cases not controlled after surgery or radiotherapy. In such situations, the endocrinologist must discern among the multiple medical treatments available to control hypercortisolism [28]. Currently, there are ketoconazole, cabergoline, mifepristone, metyrapone, and pasireotide, but an adequate knowledge on new drug treatments may be needed [29]. New drugs have been approved for treating CD, among which pasireotide which is a novel somatostatin receptor ligand that leads to normalization of cortisol levels in 20% of the patients. However, 50% of the patients develop serious side effects, such as hyperglycemia. Cabergoline was used with variable results, as well as alternative therapies focused on adrenal steroidogenesis, such as ketoconazole, metyrapone, osilodrostat, mitotane, etomidate, levoketoconazole, or targeting the peripheral glucocorticoid receptor as mifepristone. A clear knowledge of the different mechanisms of action, limitations, and even risk of such drugs become mandatory. The clinical endocrinologist role starts with the suspicion and diagnosis of the disease, and follows with the overall supervision of the treatment either surgical or medical, plus control of complications. A life-long follow up be mandatory. The PTCOE in charge of the patient may incorporate expert radiotherapists either as a part of the unit or external to treat patients not controlled by surgery or medical treatment. The outcome of radiotherapy is variable and usually takes several years. In previous years, highly focused radiation was performed by stereotactic procedures such as gamma knife or proton beam before remission of the disease was attained [30].

For neurosurgeons, expertise is based on excellent previous training and a high workload [31]. The situation is less defined for clinical endocrinologists but intuitively seems similar. It is evident that when most cases see a professional, the outcome is better. For Cushing’s disease, the problem is greater because the number of affected patients is 5 relatively small. Then, modifying the quotient between the number of specialists and the number of patients may only occur by reducing the number of specialists. That is impossible for neurosurgeons because one or a maximum of two perform such complex interventions in many hospitals. In addition, clinical endocrinologist takes care of many other pituitary diseases apart from adenomas. Therefore, it is needed to change the current scheme of experts from different divisions collaborating on a given patient to a new one denominated PTCOE that will be beneficial by definition and operate as a structured team with pre-determined meetings and fixed protocols [3]. The PTCOE is externally accredited because the periodic evaluation for outcomes performed by the accreditation commission will force to increase quality. Another option is to transform the PTCOE into a Regionalized Pituitary Tumors Center of Excellence that does not serve the inhabitants of a reduced area; instead, it serves a region with 2.5–5 million inhabitants (Fig. 1). The structure concentrates on several expert surgeons and clinical endocrinologists and attends to many patients to increase the workload. For external endocrine units to send their cases or the most difficult cases to such a regionalized structure would be better for the patient and cust-benefit the health system [3, 32].

**Fig. 1:**
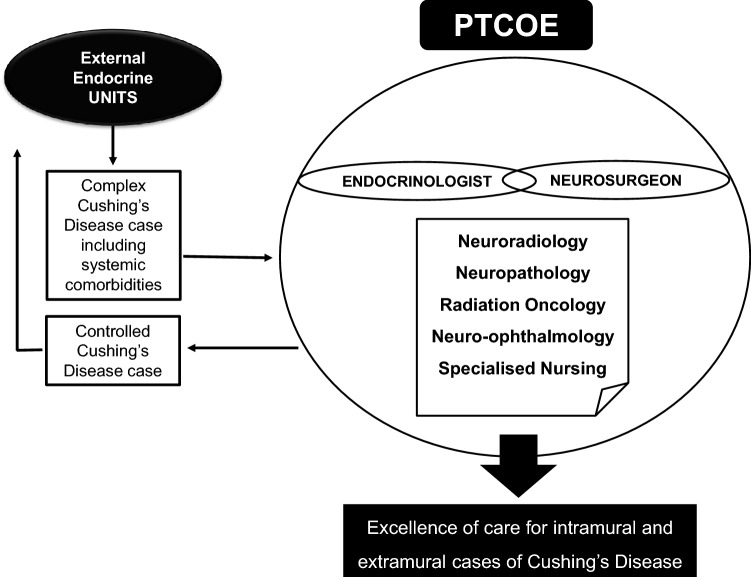
Scheme of organization of a Pituitary Tumors Center of Excellence (PTCOE). Clinical neuroendocrinologists and experienced neurosurgeons lead a multispecialist team in a structured organization with validated outcomes. The PTCOE needs to manage the intramural patients but can also give support to external endocrine units on a networking procedure

## Declarations

### Research involving human and animal participants

There is not involvement of persons or animals.
